# Imaging and clinical predictors of acute constipation in patients with acute ischemic stroke

**DOI:** 10.3389/fnins.2023.1263693

**Published:** 2023-09-13

**Authors:** I Joon Han, Ji-Eun Lee, Ha-Na Song, In-Young Baek, Jongun Choi, Jong-Won Chung, Oh Young Bang, Gyeong-Moon Kim, Woo-Keun Seo

**Affiliations:** ^1^Sungkyunkwan University School of Medicine, Seoul, Republic of Korea; ^2^Department of Neurology, Samsung Medical Center, Sungkyunkwan University School of Medicine, Seoul, Republic of Korea; ^3^Department of Digital Health, Samsung Advanced Institute for Health Sciences and Technology, Sungkyunkwan University, Seoul, Republic of Korea

**Keywords:** stroke, constipation, diffusion magnetic resonance imaging, voxel-wise lesion symptom mapping, thromboembolism

## Abstract

**Background:**

Constipation symptoms are highly prevalent in acute ischemic stroke, but the clinical and neuroimaging predictors are unknown. This study aimed to identify lesions and clinical features associated with acute constipation.

**Methods:**

Data from patients with acute ischemic stroke registered in a hospital-based stroke registry between January 2018 and December 2019 were analyzed. Clinical, laboratory, and imaging features were examined for associations with acute constipation. Using the topographic lesion on diffusion-weighted images, multivariate support vector regression-based lesion-symptom mapping (SVR-LSM) was conducted and compared between the non-constipation and acute constipation groups.

**Results:**

A total of 256 patients (mean age 67 years, men: 64%) were included. Acute constipation was noted in 81 patients (32%). Initial stroke severity, represented by initial National Institutes of Health and Stroke Scale (NIHSS) scores, was associated with acute constipation. Laboratory parameters, including fibrin degradation products (FDP), fibrinogen, D-dimer, lipoprotein (a), and free fatty acid levels, also showed statistically significant differences between the non-constipation and constipation groups. FDP, D-dimer, and free fatty acid levels were independently associated with acute constipation in the logistic regression model after adjusting for initial NIHSS scores and potassium levels. SVR-LSM revealed that bilateral lesions in the precentral gyrus, insula, opercular part of the inferior frontal gyrus, the inferior parietal lobule, and lesions in the right middle frontal gyrus were significantly associated with acute constipation. The results were consistent after controlling for the initial NIHSS scores and poststroke potassium levels. When cardioembolic stroke subjects were excluded, the right insular and prefrontal cortex lesions lost their association with acute constipation.

**Conclusion:**

Acute constipation symptoms after acute ischemic stroke are mainly related to bilateral lesions in the insula, precentral gyrus, postcentral gyrus, and inferior parietal lobule. Clinically important predictors of acute constipation include initial neurological severity and thromboembolic markers of stroke.

## Introduction

Constipation occurs in 51% of patients with acute ischemic stroke. Ischemic hemiplegia is strongly linked to constipation, (Bracci et al., 2007; Li et al., 2017) while poor stroke outcome is expected to be linked to constipation (Su et al., 2009). Despite its importance for patients’ quality of life, the mechanism of patient’s constipation after stroke is still unclear. Proposed causes of constipation in patients with stroke include immobilization, dehydration, reduced consciousness, and drug side effects (Camara-Lemarroy et al., 2014). Additionally, patients, diagnosed as stroke, with constipation had delayed colon transit time, suggesting decreased gastrointestinal (GI) motility, particularly colonic contractility, which may contribute to their constipation (Lim et al., 2012).

By categorizing lesions by brain region, previous studies have attempted to explain the association between lesion location and constipation in patients with acute ischemic stroke. Constipation and Bristol scale scores of patients with suprapontine lesions and pontine lesions were similar upon comparison (Su et al., 2009). Another study reported that *de novo* constipation was not significantly associated with any particular side of the brain hemisphere, and hypothesized that defecation is controlled bilaterally with having a dominant hemisphere, analogous to swallowing (Bracci et al., 2007). No study has attempted to identify constipation-related lesions voxel-wise (a narrower view), which may explain the lack of evidence on significant associations between lesion location and defecatory problems. Laboratory parameters of patients with acute ischemic stroke related to constipation are also unknown.

Apart from the relationship between stroke and constipation, several studies have been conducted to understand the physiology of defecation. The intrinsic enteric nervous system controls colon motility, as does the extrinsic autonomic nervous system. Parasympathetic nerve activity increases GI contraction, local blood flow, and intestinal secretion, which also influences defecation (Winge et al., 2003). Another aspect of defecation is the storage of fecal matter in the rectum and the control of the sphincter. As feces accumulate, rectal pressure rises and sensory information is relayed to the brain. When it’s time to defecate, the external sphincter relaxes and the colon and rectum peristalsis cause defecation. During this process, the relaxation of the internal sphincter is induced by increased rectal pressure (Brading and Ramalingam, 2006).

The enteric nervous system communicates with the central nervous system through the autonomic nervous system; however, in addition to the spinal cord and autonomic brainstem nuclei, higher brain regions are also expected to be involved in the interaction with the gut. Functional connectivity studies suggest differences in nodal characteristics, connectivity, and gray matter volume in the cerebrum in patients with functional constipation compared to healthy controls. These differences are observed particularly in regions such as the insula, anterior cingulate cortex, thalamus, and the precentral gyrus (Duan et al., 2021; Peihong et al., 2021; Yin et al., 2021; Jia et al., 2022). It was also revealed that anorectal stimulation causes bilateral activation of various cerebral regions, including the primary and secondary somatosensory, insular, sensory association, periorbital, anterior cingulate, and prefrontal cortices (Hobday et al., 2001). Therefore, understanding the role of cortical regions in bowel control would help explain constipation mechanisms.

Overall, this study aimed to identify the clinical variables and lesions related to acute constipation symptoms in patients with acute ischemic stroke. Clinical information, including laboratory parameters and the neurologic function of patients, were collected retrospectively. To map the association between cerebral regions and acute defecatory problems in a voxel-wise manner, voxel-wise lesion-symptom mapping was applied. Multivariate support vector regression-based lesion-symptom mapping (SVR-LSM), a promising method to apply lesion-symptom mapping (DeMarco and Turkeltaub, 2018), was utilized to analyze lesion data.

## Materials and methods

### Study design and participants

This retrospective study utilized data from the Samsung Medical Center Stroke Registry (SMC stroke registry), a tertiary hospital-based prospective stroke registry embedded in the electronic health recording system. The data pertained to the period between January 2018 and December 2019. Subjects admitted for acute ischemic stroke via the emergency room (ER) and whose diffusion-weighted imaging (DWI) files were available were included. The exclusion criteria were as follows: (1) no consent for accessing personal information, (2) diagnosed as a transient ischemic attack, small vessel occlusion, or incompletely evaluated, (3) prescribed drugs that affect GI motility (calcium channel blockers, diuretics, opioids, prokinetics, quetiapine, oral supplementary iron, dopamine receptor agonists), (4) defecation through colostomy, (5) surgery or intervention during hospitalization, (6) history of GI surgery, (7) baseline constipation, and (8) acute constipation not evaluable due to insufficient information or short hospitalization period. Lacunar infarction cases were excluded (exclusion criterion 2) because they are expected to exhibit large influences on motor function with minimal lesion effects. The study protocol for the SMC stroke registry was approved by the institutional review board of the SMC (IRB no. 2016-08-064) and all participants or their guardians provided informed consent. The study protocol for this study was also approved by the institutional review board of the SMC (IRB no. 2022-05-007). No additional informed consent was required as this was a retrospective study and no study-related adverse effects were expected. The data of this study is not available in public.

### Clinical data acquisition and analysis

#### Clinical data acquisition

The baseline characteristics of subjects, including age, sex, smoking status, alcohol consumption, stroke classification, modified Rankin Scale (mRS) and National Institutes of Health Stroke Scale (NIHSS) scores, past medical history, and laboratory examination data, were obtained from the SMC stroke registry. Subjects’ prescriptions, nursery charts, input and output (I/O) records, surgical history, and laboratory examination data not included in the registry were reviewed through Electronic Medical Records (EMR) to identify acute constipation symptoms and acquire additional information for exclusion and analysis.

#### General center protocol for patients with acute ischemic stroke and identification of acute constipation cases

Patients with acute ischemic stroke are hospitalized in the initial stroke unit for 2 to 3 days, and ambulation is limited during the period as a routine practice at the center. Antihypertensives are also limited during the first 3 days after the stroke. Acute constipation was identified in patients who consumed laxatives or were prescribed them but refused to take laxatives within 2 weeks after an acute ischemic stroke. As the Rome IV criteria for functional constipation requires evaluation for at least 6 months, prescription records were considered to be reliable indicators of acute constipation symptoms (Camara-Lemarroy et al., 2014) Laxatives were generally prescribed on the fourth day without defecation.

#### Statistical analysis

Statistical analysis of clinical data was implemented through RStudio using R 3.6.3. Descriptive statistics of the acquired clinical data are presented as mean and standard deviation, or frequency and percentage, as appropriate. The Kolmogorov–Smirnov test was used to confirm the normality of continuous variables. Two-sample *t*-tests were performed for variables that satisfied normality, while Wilcoxon rank sum tests were performed for variables with a non-normal distribution among constipation and non-constipation groups. Missing data were excluded during analysis. Chi-square analysis was performed for categorical variables. If any of the cells in the contingency table displayed a value below 5, Fisher’s exact test was performed instead. To evaluate whether each of the significant variables was independently related to constipation, logistic regression analysis was performed. Unadjusted odds ratios (OR), 95% confidence intervals (95% CI), and *P*-values were obtained from models using a single independent variable; adjusted OR, 95% CI, and *P*-values were obtained from a model that involved the initial NIHSS scores, potassium levels, and each of the following laboratory parameters: FDP, fibrinogen, D-dimer, lipoprotein (a), and free fatty acid levels.

#### Image data acquisition and structural data processing

Diffusion-weighted imaging images were acquired at 3T using a Philips Achieva instrument (manufacturer: Philips; repetition time: 3.0 s; echo tune: 81–88 ms; 22 slices; matrix size: 256 × 256; pixel spacing: 0.94 × 0.94; spacing between the slices: 6.5 mm); *b* = 0 and *b* = 1,000 images were obtained. DWI and apparent diffusion coefficient (ADC) Digital Imaging and Communications in Medicine (DICOM) data for the subjects were de-identified and converted to the Neuroimaging Informatics Technology Initiative (NIFTI) format using the dcm2nii tool of the MRIcron program (Li et al., 2016). The DWI *b* = 1,000 images and ADC images were applied to perform lesion segmentation and lesion volume calculation using the validated U-Net segmentation model (Kim et al., 2019). Images were then normalized using the clinical toolbox^1^ provided in the Statistical Parametrical Mapping (SPM) 12 software.^2^ The ch2bet.nii template file provided in the MRIcron software was used as the template.

#### Multivariate lesion-symptom mapping

Multivariate lesion-symptom mapping was conducted using support vector regression (SVR). SVR was implemented using the MATLAB SVR lesion-symptom mapping toolbox^3^ (DeMarco and Turkeltaub, 2018). The analysis was limited to voxels, showing a minimal lesion overlap of *n* = 5 voxels. The primary analysis was crude without any covariates. Then, analysis was performed, including initial NIHSS and potassium as covariates, applied to both the behavioral score and lesion data. Sensitivity analysis excluding patients with cardioembolic stroke was also implemented. Lesion volume correction adjusting for both the behavior and lesion data were performed for the entire analysis. One thousand permutations were computed, and the critical values at the voxel- and cluster-wise levels were both fixed at *P* = 0.05. The results of the analysis were visualized using the xjview10 toolbox^4^ based on SPM12. The locations of clusters were identified and organized based on voxel size and intensity.

The journal conformed to the STROBE guidelines for observational studies.^5^

## Results

### General characteristics and clinical features of patients with or without constipation

**FIGURE 1:**
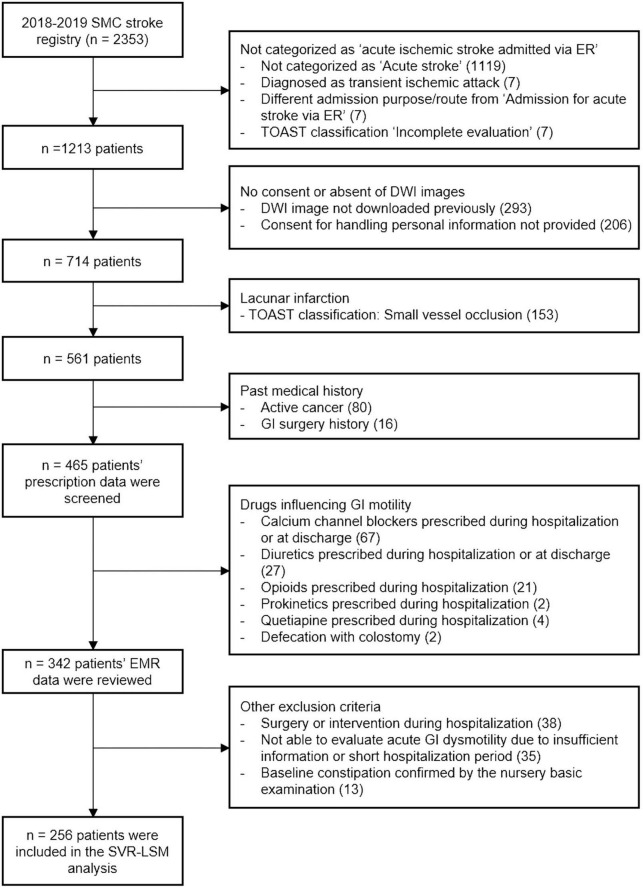
Flowchart of inclusion and exclusion criteria. DWI, diffusion-weighted images; EMR, electronic medical records; ER, emergency room; TOAST, Trail of ORG 10,172 in Acute Stroke Treatment; GI, gastrointestinal; SMC, Samsung Medical Center; SVR-LSM, support vector regression-based lesion-symptom mapping.

Among 2,353 subjects registered in the SMC stroke registry between January 2018 and December 2019, 256 patients with acute ischemic stroke were selected by the process shown in Figure 1. Among the 256 patients (mean age 67.0 years, 91 women), 39.84% had a large artery atherosclerosis stroke, 25.00% had a cardioembolic stroke, and the rest of the patients were classified into other, cryptogenic, or unclassified categories according to the Trail of ORG 10,172 in Acute Stroke Treatment (TOAST) classification (Table 1; Adams et al., 1993) Statistically significant sex-based difference of the proportion of constipation in patients with stroke was not observed (Table 1).

**TABLE 1:** Descriptive statistics of clinical variables in subjects with or without acute constipation symptoms.

Variable	Category	Non-constipation (*N* = 175)	Constipation (*N* = 81)	Total (*N* = 256)	*P*-value
Age, years		66.1 ± 12.7	68.9 ± 12.3	67.0 ± 12.6	0.104
Sex	Female	56 (32.00%)	35 (43.21%)	91 (35.55%)	0.081
	Male	119 (68.00%)	46 (56.79%)	165 (64.45%)	
Smoking status	Never smoker	110 (62.86%)	49 (60.49%)	159 (62.11%)	0.757
	Ex-smoker	35 (20.00%)	15 (18.52%)	50 (19.53%)	
	Current smoker	30 (17.14%)	17 (20.99%)	47 (18.36%)	
Alcohol consumption, bottles per week		0.8 ± 2.0	1.0 ± 3.1	0.9 ± 2.4	0.521
Hypertension		114 (65.14%)	59 (72.84%)	173 (67.58%)	0.221
Diabetes mellitus		50 (28.57%)	29 (35.80%)	79 (30.86%)	0.244
Dyslipidemia		112 (64.00%)	55 (67.90%)	167 (65.23%)	0.542
Previous stroke or TIA*		46 (26.29%)	23 (28.40%)	69 (26.95%)	0.724
Congestive heart failure		3 (1.71%)	2 (2.47%)	5 (1.95%)	0.653
Peripheral arterial occlusive disease		5 (2.86%)	0 (0.00%)	5 (1.95%)	0.183
Coronary artery disease		20 (11.43%)	6 (7.41%)	26 (10.16%)	0.373
Atrial fibrillation		33 (18.86%)	14 (17.28%)	47 (18.36%)	0.995
Fibrin degradation products, μg/mL (*N* = 244)		3.34 ± 3.71	11.2 ± 31.4	5.9 ± 18.3	0.003**
Fasting glucose, mg/dL (*N* = 250)		125.6 ± 48.5	115.1 ± 28.4	122.2 ± 43.4	0.638
HbA1c,% (*N* = 192)		6.3 ± 1.1	6.2 ± 1.1	6.3 ± 1.1	0.716
Uric acid, mg/dL (*N* = 248)		5.2 ± 1.4	5.1 ± 1.5	5.2 ± 1.4	0.324
Prothrombin time, International Normalized Ratio (INR) (*N* = 255)		1.0 ± 0.2	1.0 ± 0.1	1.0 ± 0.2	0.484
Activated partial thromboplastin clotting time, sec (*N* = 255)		35.03 ± 4.75	33.90 ± 4.45	34.67 ± 4.68	0.059
D-dimer, μg/mL (*N* = 254)		0.7 ± 0.9	1.6 ± 3.2	1.0 ± 1.9	0.002**
Cholesterol, mg/dL		174.4 ± 45.8	171.6 ± 46.7	173.5 ± 46.0	0.660
Triglyceride, mg/dL		146.7 ± 115.5	149.1 ± 116.9	147.4 ± 115.7	0.860
HDL^†^ cholesterol, mg/dL		49.1 ± 14.5	49.8 ± 12.7	49.3 ± 13.9	0.315
LDL^‡^ cholesterol, mg/dL		114.3 ± 39.8	109.9 ± 41.9	112.9 ± 40.4	0.414
Fibrinogen, mg/dL (*N* = 252)		325.3 ± 68.3	345.4 ± 72.5	331.8 ± 70.2	0.024*
Lipoprotein (a), mg/dL (*N* = 246)		21.7 ± 22.7	23.7 ± 17.4	22.3 ± 21.1	0.032*
Free fatty acid, mmol/L (*N* = 242)		0.650 ± 0.347	0.798 ± 0.379	0.697 ± 0.363	0.003**
Phosphate, mg/dL (*N* = 254)		3.3 ± 0.6	3.3 ± 0.6	3.3 ± 0.6	0.857
Sodium, mEq/L		140.1 ± 4.7	140.0 ± 2.6	140.1 ± 4.1	0.307
Potassium, mEq/L		4.3 ± 0.4	4.2 ± 0.4	4.3 ± 0.4	0.058
Chloride, mEq/L		103.4 ± 2.7	103.5 ± 3.0	103.4 ± 2.8	0.810
Magnesium, mg/dL (*N* = 252)		2.1 ± 0.2	2.0 ± 0.2	2.1 ± 0.2	0.315
Ionized calcium, mmol/L (*N* = 252)		1.19 ± 0.10	1.19 ± 0.05	1.19 ± 0.09	0.494
Initial NIHSS^§^		3.3 ± 3.5	5.5 ± 4.4	4.0 ± 4.0	<0.001***
Previous modified Rankin Scale (mRS)		0.1 ± 0.5	0.3 ± 0.9	0.2 ± 0.6	0.154
mRS at 7 days or discharge		1.5 ± 1.2	2.5 ± 1.3	1.8 ± 1.3	0.001***
NIHSS at 7 days or discharge		2.8 ± 5.9	4.2 ± 3.8	3.2 ± 5.3	<0.001***
TOAST^||^ classification	Large artery atherosclerosis	63 (36.00%)	39 (48.15%)	102 (39.84%)	0.193
	Cardioembolic	45 (25.71%)	19 (23.46%)	64 (25.00%)	
	Other	32 (18.29%)	8 (9.88%)	40 (15.63%)	
	Cryptogenic/Unclassified (2 or no probable)	35 (20.00%)	15 (18.52%)	50 (19.53%)	

Mean and standard deviation are provided for continuous variables, and frequency and proportion are provided for categorical variables. The *P*-values are calculated using either the chi-square test, Fisher’s exact test, two-sample *t*-test, or Wilcoxon rank sum test, as appropriate (*: *P* < 0.05, **: *P* < 0.01, ***: *P* < 0.001).

*TIA, transient ischemic attack.

^†^HDL, high-density lipoprotein.

^‡^LDL, low-density lipoprotein.

^§^ NIHSS, National Institutes of Health Stroke Scale.

^||^ TOAST, Trial of ORG 10,172 in Acute Stroke Treatment.

Acute constipation was noted in 81 patients (31.6%). The initial NIHSS score was significantly higher in the constipation group than in the non-constipation group. There was a statistically significant difference in the NIHSS score and mRS score at 7 days or discharge, while previous mRS scores did not vary significantly, indicating that the severity of the latest stroke is an important factor influencing acute constipation symptoms. In terms of the laboratory data, fibrin degradation products (FDP), D-dimer, fibrinogen, lipoprotein (a), and free fatty acid levels were significantly higher in the constipation group. Other demographic, clinical, and laboratory parameters were not significantly different between constipation and non-constipation groups (Table 1 and Supplementary Table 1).

The initial NIHSS was independently associated with acute constipation in logistic regression analysis (OR: 1.027, 95% CI: 1.011–1.042; Table 2), indicating that the differences in the suggested laboratory data were dependent on stroke severity. FDP (OR: 1.004, 95% CI: 1.001–1.007), D-dimer (OR: 1.036, 95% CI: 1.007–1.067), and free fatty acid levels (OR: 1.211, 95% CI: 1.035–1.416) were also independently associated with acute constipation in the adjusted model (Table 2).

**TABLE 2:** Logistic regression analysis of the association between fibrin degradation products (FDP), D-dimer, fibrinogen, lipoprotein (a), free fatty acid levels, initial National Institutes of Health Stroke Scale (NIHSS) score, and poststroke potassium level, and acute constipation.

	Unadjusted OR* (95% CI^†^)	*P*-value	Adjusted OR (95% CI)	*P*-value
Fibrin degradation product, μg/mL	1.005 (1.002, 1.008)	0.001**	1.004 (1.001, 1.007)	0.012*
Fibrinogen, mg/dL	1.001 (1.000, 1.002)	0.116	1.000 (1.000, 1.001)	0.366
D-dimer, μg/mL	1.046 (1.016, 1.077)	0.003**	1.036 (1.007, 1.067)	0.016*
Lipoprotein (a), mg/dL	1.001 (0.998, 1.004)	0.584	1.001 (0.998, 1.004)	0.417
Free fatty acid, mmol/L	1.276 (1.088, 1.496)	0.003**	1.211 (1.035, 1.416)	0.017**
Initial NIHSS^‡^	1.034 (1.019, 1.049)	<0.001***	1.033 (1.018, 1.048)	<0.001***
Potassium, mEq/L	0.888 (0.765, 1.030)	0.118	0.920 (0.796, 1.063)	0.258

The unadjusted odds ratio (OR) was calculated through logistic regression with a single variable; adjusted OR was calculated through logistic regression with each variable, and the initial NIHSS and potassium levels were included. The adjusted OR of the initial NIHSS and potassium levels indicates the OR from the model containing both the initial NIHSS and potassium levels. (*: *P* < 0.05, **: *P* < 0.01, ***: *P* < 0.001).

*OR, odds ratio.

^†^CI, confidence interval.

^‡^NIHSS, National Institutes of Health Stroke Scale.

### Multivariate lesion-symptom mapping

**FIGURE 2:**
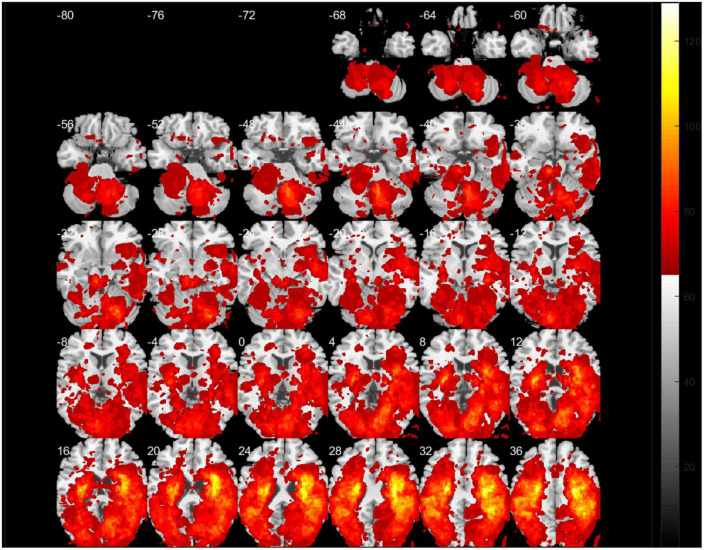
A lesion overlap map of subjects included in the support vector regression-based lesion-symptom mapping (SVR-LSM) analysis (*N* = 230). White numbers above the slices indicate the *z*-axis Montreal Neurologic Institute (MNI) coordinates of each transverse section. The spacing between each transverse section is fixed at 4 mm.

**FIGURE 3:**
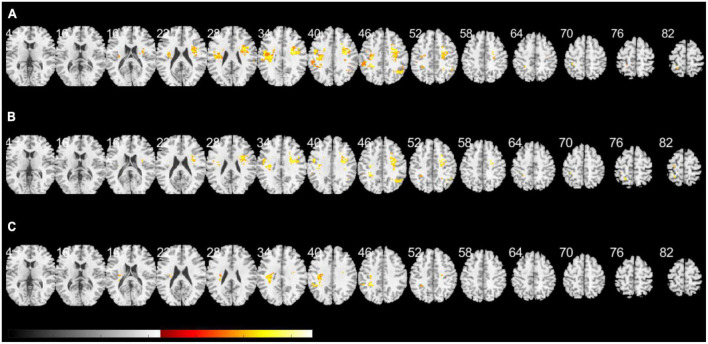
A voxel-wise threshold beta map of the support vector regression-based lesion-symptom mapping (SVR-LSM) analysis on acute constipation. **(A)** Crude analysis. **(B)** Analysis regressing out initial National Institutes of Health and Stroke Scale (NIHSS) and potassium levels as behavioral covariates. **(C)** Sensitivity analysis excluding cardioembolic stroke. Voxels satisfying *P* < 0.05 are shown. White numbers above the slices indicate the *z*-axis Montreal Neurologic Institute (MNI) coordinates of each transverse section. The spacing between each transverse section is fixed at 6 mm. Brain regions under z-axis MNI coordinate 4 did not show any significant lesions.

Figure 2 presents a lesion overlap map including all subjects (*n* = 230); 26 subjects were excluded due to the absence of voxels inside the minimum lesion cutoff mask. The regions involved in middle cerebral artery strokes showed the highest overlap, but most of the brain regions were covered by the lesion overlap of the 230 subjects. Figure 3A illustrates a voxel-wise thresholded beta map that shows voxels that satisfy *P* < 0.05 in the crude analysis. The bilateral lesions were related to acute constipation symptoms. Table 3 describes clusters with all voxels satisfying *P* < 0.05 and voxel size > 100 in the crude analysis. The centers of clusters in the right cerebrum were located in the precentral gyrus, angular gyrus, and supramarginal gyrus. The location of peak intensity included the right insula, angular gyrus, and supramarginal gyrus. The biggest cluster also involved the opercular part of the inferior frontal gyrus, the middle frontal gyrus, the postcentral gyrus, and the superior frontal gyrus, which each contained more than 500 voxels. The center and peak intensity of clusters in the left cerebrum were both located in the precentral gyrus and the superior parietal lobule. The biggest cluster also contained the inferior parietal lobule, postcentral gyrus, and insula; it also contained more than 500 voxels.

**TABLE 3:** Features of clusters in the crude SVR-LSM analysis.

	Cluster number	Number of voxels	Anatomical location of the center of gravity	Center MNI* coordinates	Anatomical location of peak intensity	Peak MNI coordinates	Anatomical locations involved in the cluster (>500)
Right	1	18,180	Precentral gyrus	37 −3 37	Insula	36 −1 16	Precentral gyrus Opercular part of the inferior frontal gyrus Middle frontal gyrus Postcentral gyrus Superior frontal gyrus Insula
	2	2,146	Angular gyrus	52 −62 47	Angular gyrus	43 −63 44	
	3	331	Angular gyrus	30 −59 50	Angular gyrus	30 −61 48	
	4	120	Supramarginal gyrus	68 −27 27	Supramarginal gyrus	68 −22 24	
	5	109	Supramarginal gyrus	48 −40 43	Supramarginal gyrus	49 −42 42	
Left	6	17,271	Precentral gyrus (postcentral in aal3v1 atlas)	−44 −21 37	Precentral gyrus	−55 −6 31	Postcentral gyrus Precentral gyrus Supramarginal gyrus Insula
	7	193	Undefined	−28 −54 81	Undefined	−28 −55 85	
	8	179	Superior parietal lobule	−31 −49 66	Superior parietal lobule	−31 −46 66	
	9	172	Undefined	−30 −45 79	Undefined	−26 −44 79	
	10	131	Undefined	−41 −41 70	Undefined	−41 −38 63	

Clusters with > 100 voxels satisfying *P* < 0.05 are shown. Results are listed in descending order of voxel size for each hemisphere. The locations of peak intensity and center of gravity are provided as brain regions and Montreal Neurological Institute (MNI) coordinates. Anatomical description from the xjview program is specified based on the Talairach Atlas Labels, and the brain regions on the Automated Anatomical Labelling atlas 3 (AAL3v1) are also provided. In case of differences between the two suggested regions, both regions are described in the table. As the anatomical location of the center and peak intensity did not fully represent anatomical regions in large clusters, anatomical locations with > 500 voxels in the cluster are additionally provided. *MNI, Montreal Neurological Institute.

Multivariate SVR-LSM analysis was conducted with the initial NIHSS and potassium levels. Potassium level, which is clinically considered to influence bowel symptoms, was applied as a covariate, though it was not significantly related to constipation in the logistic regression model. Figure 3B shows a voxel-wise thresholded beta map depicting voxels with *P* < 0.05. Frontal lobe lesions on both hemispheres were found to be significant in the voxel-wise map; the right hemisphere exhibited more dominant lesions. Clusters of voxels satisfying *P* < 0.05 are illustrated in Supplementary Table 2. Similar to the crude analysis, the precentral gyrus, middle frontal gyrus, insula, opercular part of the inferior frontal gyrus, superior frontal gyrus, and angular gyrus were included in the clusters of the right cerebrum. Clusters of the left cerebrum included the precentral gyrus, postcentral gyrus, inferior parietal lobule, and superior parietal lobule.

As parameters related to thromboembolic events, such as FDP, fibrinogen, D-dimer, and free fatty acid levels, differed between constipation and non-constipation groups, a sensitivity analysis excluding the cardioembolic stroke subjects was applied. Initially, 192 subjects were included, but 37 subjects were excluded for having no voxels inside the minimum lesion cutoff mask. Although fewer subjects were included, most of the brain regions were covered on the lesion overlap map, as in the crude analysis (Supplementary Figure 1). However, compared to the crude analysis and the analysis with covariates, lesions in the left cerebrum were more dominant in the voxel-wise thresholded beta map (Figure 3C). Only one cluster with the highest intensity in the left cerebrum and the left insular was significant at cluster-wise *P* < 0.05. This cluster included the inferior parietal lobule, postcentral gyrus, precentral gyrus, and insula and corresponded with the locations suggested in Supplementary Table 3.

## Discussion

In this study, the relationship between clinical and topographic characteristics and acute constipation symptoms was analyzed in patients with acute ischemic stroke. Initial neurological severity and markers of thromboembolism were identified as independent predictors of acute constipation. Regarding topographic features, bilateral lesions in the precentral gyrus, postcentral gyrus, insula, opercular part of the inferior frontal gyrus, the inferior parietal lobule, and lesions in the middle and superior frontal gyrus of the right hemisphere, and the superior parietal lobule of the left hemisphere, were found to be associated with acute constipation symptoms. Especially, lesions in the insula, precentral gyrus, postcentral gyrus, and inferior parietal lobule showed significant association with constipation both in crude and sensitivity analyses.

Considering that initial NIHSS scores and mRS and NIHSS scores at 7 days or discharge were significantly associated with constipation, mobility seems to play an important role in acute defecatory problems. The fact that lesions in the precentral gyrus, essential for motor function, were associated with acute constipation also supports this idea. However, the symptoms in the constipation group were not simply due to the limitation of mobility, as the lesions were significantly related to constipation after adjusting for the initial NIHSS scores. One possible component influencing constipation symptoms may be visceral sensory perception. During rectal distention, the right anterior insula, left insula, bilateral thalamus, left and right postcentral gyrus, and the right parietal lobule are activated, and afferent signals from the anterior insula increase the drive to defecate in healthy adults (Halani et al., 2020) Therefore, patients with stroke in the frontal lobe, including the anterior insula, may have acute defecatory problems and feel less desire to defecate. Additionally, anal and rectal sensations trigger cortical activation of regions involved with spatial discrimination, attention, and affect (Hobday et al., 2001). This explains the correlation of parietal lobe lesions with acute constipation. The lesions found significant in our study were located in regions related to anorectal sensation, implying that the acute constipation symptoms of patients with stroke are influenced by aberrances in accepting and interpreting visceral afferent signals from the anal canal and rectum. The fairly symmetric distribution of the associated lesions also confirms the existing hypothesis that cortical control of the defecation center might be located bilaterally (Bracci et al., 2007; Kasaraneni and Hayes, 2014). This may be related to the bilateral activation of cortical areas by rectal stimulation (Hobday et al., 2001). In summary, acute constipation symptoms are related to regions associated with both motor and visceral sensory function.

Although this study explored the relationship between lesions and acute constipation, the results correspond with structural and functional brain studies about chronic functional constipation. Decreased gray matter volumes in the insula, middle frontal gyrus, and anterior cingulate cortex, and decreased connectivity between the insula and middle frontal gyrus and between the anterior cingulate cortex and right middle frontal gyrus were suggested as features related to functional constipation (Jia et al., 2022). The results of our study imply that damage in the insula and middle frontal gyrus correlate not only with chronic and functional problems in defecation but also with acute defecation problems.

Analysis of laboratory parameters revealed that there was a significant difference in the distribution of FDP, fibrinogen, D-dimer, lipoprotein (a), and free fatty acid levels between constipation and non-constipation groups. Logistic regression also revealed an association between acute constipation and FDP, D-dimer, free fatty acid levels, and initial NIHSS scores. Elevated FDP, fibrinogen, and D-dimer levels are associated with a higher risk of thromboembolism (Moresco et al., 2006). The free fatty acid is also related to thrombin generation, as it allosterically inhibits zinc from binding to albumin (Lagrange et al., 2017; Coverdale et al., 2019). The zinc displaced from albumin binds to histidine-rich glycoprotein, which could induce a procoagulatory effect through interaction with heparin and heparin sulfate (Kassaar et al., 2015). The fact that FDP, D-dimer, and free fatty acid levels were associated with acute constipation in the logistic regression model including initial NIHSS scores implies that acute constipation may be related to thromboembolic status regardless of stroke severity. Underlying mechanisms, such as the risk of hidden mesenteric embolism, should be further investigated.

As cardioembolic stroke frequently involves the middle cerebral artery region, it was important to confirm whether the result from the crude analysis was independent of cardioembolic stroke. We performed SVR-LSM analysis after excluding subjects with cardioembolic stroke. The result of this sensitivity analysis differed from the crude analysis and analysis with covariates in aspects of cluster size and the dominant hemisphere. There are two possible reasons for this result. Cardioembolic stroke frequently occurs in the right hemisphere (Park et al., 2014), is the most severe subtype (Arboix and Alió, 2010), and its initial neurological severity is associated with acute constipation. This may be a cause of right-hemisphere dominance in the crude analysis and the weakness of significance in the sensitivity analysis. Additionally, these differences may be due to the small sample size and smaller regions meeting the minimum lesion overlap criterion for the analysis. The area meeting the minimum lesion overlap criterion (*n* = 5) in the right cerebrum was smaller than that in the left cerebrum in the sensitivity analysis, which may explain why larger areas were significant in the left cerebrum. The lesions associated with constipation in the sensitivity analysis, including the left insula, inferior parietal lobule, postcentral gyrus, and right precentral gyrus, corresponded to the lesions identified in the crude analysis, implying that the association between lesion location and constipation is not simply dependent on cardiac embolism.

Our study has several limitations. Primarily, as this study was conducted retrospectively, the case definition of acute constipation was mostly dependent on the prescriptions issued during the subjects’ hospitalization. Each subject had different hospitalization periods, which may have caused decreased accuracy in identifying acute constipation. To minimize the issue, patients with short hospitalization periods (<4 days) were excluded, and patients with long hospitalization periods were only inspected for the first 2 weeks. Although a previous study on acute constipation symptoms defined “new-onset constipation” as constipation that occurred 1 month after stroke (Su et al., 2009), we were unable to apply this definition because most of the patients were discharged earlier unless they were transferred to the rehabilitation unit. For constipation cases, laxatives were usually prescribed 4–7 days after the stroke. Since the case definition was dependent on the center’s prescription protocol of laxatives, our study has limitations in guaranteeing external validity. In addition to limitations in case definition, there are limitations in covariate selection. The NIHSS is a representative stroke assessment scale that evaluates categories such as level of consciousness, eye movement, sensory function, facial palsy, language, and inattention, in addition to motor functions. Therefore, it would have been more appropriate to utilize the initial mRS score as a covariate to rule out the effect of ambulation on constipation. However, as the SMC stroke registry did not include information about the initial mRS score, the NIHSS score was selected.

## Conclusion

In conclusion, our study investigated clinical and imaging predictors related to acute constipation in patients with acute ischemic stroke. The right middle frontal gyrus, left superior parietal lobule, bilateral precentral gyrus, postcentral gyrus, insula, and inferior parietal lobule were found to be significantly associated with acute constipation. These findings were consistent after regressing out the initial NIHSS scores and potassium levels. Insular, precentral, postcentral, and inferior parietal lesions were significant in the sensitivity analysis. Among clinical parameters, initial neurological severity and thromboembolic markers were significantly associated with acute constipation. Patients with high thromboembolic risk and the severe neurological deficits or lesions mentioned above should be more carefully examined for defecatory problems.

## Data availability statement

The raw data supporting the conclusions of this article will be made available by the authors, without undue reservation.

## Ethics statement

The studies involving humans were approved by the Samsung Medical Center institutional review board. The studies were conducted in accordance with the local legislation and institutional requirements. The Ethics Committee/institutional review board waived the requirement of written informed consent for participation from the participants or the participants’ legal guardians/next of kin because the study was conducted retrospectively among subjects in the prospective SMC stroke registry. There is minimal chance of harm when informed consent is waived. Also, a large portion of subjects are not currently visiting the out-patient clinic, which makes it difficult to acquire informed consent.

## Author contributions

IJH: Conceptualization, Investigation, Visualization, Writing—original draft, Writing—review and editing, Formal-Analysis. J-EL: Data curation, Formal-Analysis, Software, Validation, Writing—review and editing. H-NS: Data curation, Software, Writing—review and editing. I-YB: Data curation, Methodology, Validation, Writing—review and editing. JC: Writing—review and editing. J-WC: Resources, Supervision, Writing—review and editing. OYB: Resources, Supervision, Writing—review and editing. G-MK: Resources, Supervision, Writing—review and editing. W-KS: Writing—review and editing, Conceptualization, Data curation, Funding acquisition, Methodology, Project administration, Resources, Supervision, Validation.

## Footnotes

1
https://www.nitrc.org/projects/clinicaltbx/

2
https://www.fil.ion.ucl.ac.uk/spm/software/spm12/

3
github.com/atdemarco/svrlsmgui

4
https://www.alivelearn.net/xjview

5
http://www.strobe-statement.org/
