# Supplementary material for: Imaging and clinical predictors of acute constipation in patients with acute ischemic stroke
Source: Front Neurosci. 2023 Sep 13;17:1263693. doi: 10.3389/fnins.2023.1263693 (PMC10534029; doi:10.3389/fnins.2023.1263693)
Supplement: Supplementary file 1 [file Data_Sheet_1.PDF]

Supplementary material

Supplementary Tables

Supplementary Table 1. Laboratory parameters in subjects with or without acute constipation symptoms. Mean and standard deviations are provided. The *P*-values are calculated from either two-sample *t*-tests or Wilcoxon rank sum tests according to normality

Variable	Non-constipation (N = 175)	Constipation (N = 81)	Total (N = 256)	*P*-value
Hemoglobin, g/dL	14.0 ± 1.8	13.6 ± 1.6	13.9 ± 1.8	0.075
Hematocrit, L/L	41.8 ± 5.0	40.6 ± 4.2	41.4 ± 4.8	0.052
White Blood Cell count, x10^3 cells/μL	7.4 ± 2.2	7.6 ± 2.3	7.5 ± 2.3	0.668
Platelet x10^3 cells/μL	221.0 ± 68.6	216.6 ± 66.8	219.6 ± 68.0	0.631
Erythrocyte sedimentation rate, mm/h	16.1 ± 12.6	18.9 ± 15.6	17.0 ± 15.6	0.067
C-reactive protein, mg/dL	0.4 ± 1.0	0.4 ± 1.0	0.4 ± 1.0	0.862
Homocysteine, mmol/L	13.2 ± 8.5 (Missing: 13)	13.4 ± 6.8 (Missing: 8)	13.2 ± 8.0	0.980
Apolipoprotein A, mg/dL	134.6 ± 27.8 (Missing: 8)	135.1 ± 24.9 (Missing: 3)	134.8 ± 26.9	0.895
Apolipoprotein B, mg/dL	97.6 ± 28.7 (Missing: 8)	95.5 ± 30.5 (Missing: 3)	96.9 ± 29.2	0.603

Total protein, g/dL	7.0 ± 0.5	7.0 ± 0.5	7.0 ± 0.5	0.408
Albumin, g/dL	4.4 ± 0.4	4.3 ± 0.3	4.4 ± 0.4	0.173
Total bilirubin, mg/dL	0.6 ± 0.3	0.6 ± 0.3	0.6 ± 0.3	0.536
Aspartate aminotransferase, U/L	26.5 ± 21.0	24.9 ± 8.9	26.0 ± 18.1	0.887
Alanine transaminase, U/L	23.2 ± 20.4	21.2 ± 13.2	22.6 ± 18.5	0.163
Blood urea nitrogen, mg/dL	16.5 ± 7.3	17.6 ± 8.3	16.9 ± 7.6	0.235
Creatinine, mg/dL	1.0 ± 0.6	1.6 ± 5.9	1.2 ± 3.4	0.335
eGFR[†], mL/min/1.73 m^2	84.9 ± 63.7	79.6 ± 20.3	83.2 ± 53.9	0.781

[†]eGFR: estimated glomerular filtration rate

Supplementary Table 2. Feature of clusters in the support vector regression-based lesion-symptom mapping (SVR-LSM) analysis with covariates. Clusters with >100 voxels satisfying $P < 0.05$ are shown. Results are listed in descending order of voxel size for each hemisphere. The locations of peak intensity and center of gravity are provided as brain regions and Montreal Neurological Institute (MNI) coordinates. The anatomical description from the xjview program is specified based on the Talairach Atlas Labels, and the brain regions on the Automated Anatomical Labelling atlas 3 (AAL3v1) are also provided. In case of differences in the two suggested regions, both the regions are described in the table. As the anatomical location of the center and peak intensity did not fully represent anatomical regions in large clusters, anatomical locations with >500 voxels in the cluster are additionally provided

	Cluster number	Number of voxels	Anatomical location of the center of gravity	Center MNI[†] coordinates	Anatomical location of peak intensity	Peak MNI coordinates	Anatomical locations involved in the cluster (>500)
Right	1	11,777	Middle frontal gyrus	36 -2 38	Insula	36 -1 17	Precentral gyrus<br>Middle frontal gyrus<br>Opercular part of the inferior frontal gyrus<br>Superior frontal gyrus<br>Postcentral gyrus
	2	1,458	Angular gyrus	52 -64 47	Angular gyrus	43 -63 44	Angular gyrus
	3	232	Angular gyrus	30 -59 51	Angular gyrus	30 -61 48	
Left	4	1,738	Precentral gyrus (Postcentral gyrus in aal3v1 atlas)	-54 -4 37	Precentral gyrus (Postcentral gyrus in aal3v1 atlas)	-52 -3 37	Precentral gyrus<br>Postcentral gyrus<br>Brodmann area 6

5	1,731	Undefined (sub-gyral white matter of the frontal lobe)	-39	-21	38	Precentral gyrus (Postcentral gyrus in aal3v1 atlas)	-32	-22	34	Precentral gyrus<br>Postcentral gyrus
6	1,139	Inferior parietal lobule	-36	-48	48	Inferior parietal lobule	-30	-58	47	Inferior parietal lobule
7	621	Undefined	-27	-57	79	Undefined	-28	-55	85	
8	209	Precentral gyrus	-42	-6	28	Undefined (sub-gyral white matter of the frontal lobe)	-41	-2	20	
9	166	Undefined	-31	-20	81	Undefined	-29	-22	79	
10	155	Superior parietal lobule	-31	-49	66	Superior parietal lobule	-31	-46	66	
11	154	Undefined	-31	-46	79	Undefined	-28	-44	76	
12	142	Undefined	-41	-41	70	Undefined	-38	-41	73	
13	124	Undefined	-61	-44	43	Undefined	-59	-44	40	
14	117	Undefined (sub-gyral white matter of the frontal lobe)	-26	6	39	Undefined (sub-gyral white matter of the frontal lobe)	-27	8	33	

†MNI: Montreal Neurological Institute

Supplementary Table 3. Features of clusters in the support vector regression-based lesion-symptom mapping (SVR-LSM) analysis after excluding cardioembolic stroke subjects. Clusters with >100 voxels satisfying P <0.05 are shown. Results are listed in descending order of voxel size for each hemisphere. Locations of peak intensity and center of gravity are provided as brain regions and MNI coordinates

	Cluster number	Number of voxels	Anatomical location of the center of gravity	Center MNI[†] coordinates	Anatomical location of peak intensity	Peak MNI coordinates	Anatomical locations involved in the cluster (>500)
Right	1	174	Precentral gyrus	26 -18 51	Precentral gyrus	24 -20 52	
	2	132	Undefined (sub-gyral white matter of the frontal lobe)	32 -10 35	Undefined (sub-gyral white matter of the frontal lobe)	33 -10 37	
Left	3	6,426	Postcentral gyrus (inferior parietal lobule in aal3v1 atlas)	-39 -31 38	Inferior parietal lobule	-36 -47 47	Inferior parietal lobule<br>Postcentral gyrus<br>Precentral gyrus
	4	139	Undefined (sub-lobar white matter)	-28 -9 17	Undefined (sub-lobar white matter)	-28 -10 17	
	5	119	Insula	-37 -34 19	Insula (Rolandic operculum in aal3v1 atlas)	-35 -35 19	

[†]MNI: Montreal Neurological Institute

Supplementary Figures

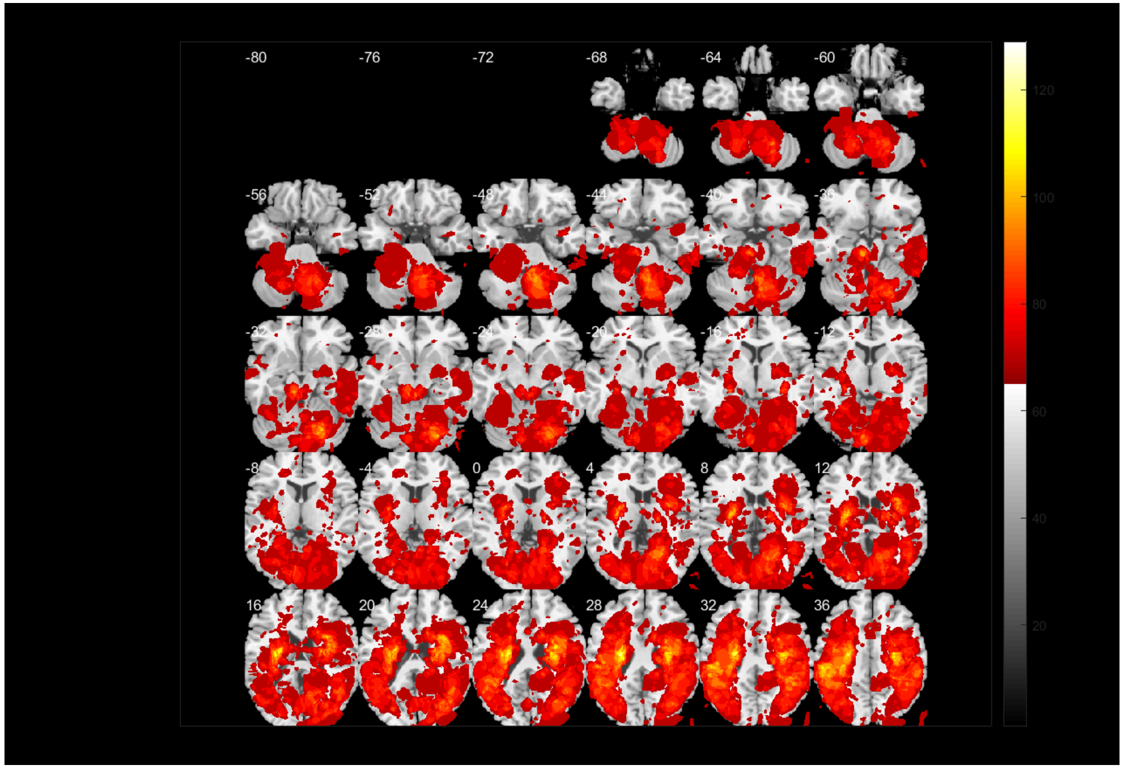

Supplementary Figure 1. A lesion overlap map of subjects included in sensitivity analysis (cardioembolic stroke subjects were excluded) (N = 155). White numbers above the slices indicate the z-axis Montreal Neurological Institute (MNI) coordinates of each transverse section. The spacing between each transverse section is fixed at 4 mm.
